# Metabolic Analysis Reveals *Cry1C* Gene Transformation Does Not Affect the Sensitivity of Rice to Rice Dwarf Virus

**DOI:** 10.3390/metabo11040209

**Published:** 2021-03-30

**Authors:** Xuefei Chang, Duo Ning, Lijuan Mao, Beibei Wang, Qi Fang, Hongwei Yao, Fang Wang, Gongyin Ye

**Affiliations:** 1 State Key Laboratory of Rice Biology & Ministry of Agriculture and Rural Affairs Key Laboratory of Molecular Biology of Crop Pathogens and Insects, Institute of Insect Sciences, Zhejiang University, Hangzhou 310058, China; changxuefei2015@163.com (X.C.); ningduo2013@163.com (D.N.); wangbei_zju@163.com (B.W.); fangqi@zju.edu.cn (Q.F.); hwyao@zju.edu.cn (H.Y.); 2 Analysis Center of Agrobiology and Environmental Sciences, Zhejiang University, Hangzhou 310058, China; mlj105@163.com

**Keywords:** rice dwarf virus, *Cry1C* rice, metabolites, free amino acids, RDV infection rates

## Abstract

Metabolomics is beginning to be used for assessing unintended changes in genetically modified (GM) crops. To investigate whether *Cry1C* gene transformation would induce metabolic changes in rice plants, and whether the metabolic changes would pose potential risks when *Cry1C* rice plants are exposed to rice dwarf virus (RDV), the metabolic profiles of *Cry1C* rice T1C-19 and its non-*Bt* parental rice MH63 under RDV-free and RDV-infected status were analyzed using gas chromatography–mass spectrometry (GC-MS). Compared to MH63 rice, slice difference was detected in T1C-19 under RDV-free conditions (less than 3%), while much more metabolites showed significant response to RDV infection in T1C-19 (15.6%) and in MH63 (5.0%). Pathway analysis showed biosynthesis of lysine, valine, leucine, and isoleucine may be affected by RDV infection in T1C-19. No significant difference in the contents of free amino acids (AAs) was found between T1C-19 and MH63 rice, and the free AA contents of the two rice plants showed similar responses to RDV infection. Furthermore, no significant differences of the RDV infection rates between T1C-19 and MH63 were detected. Our results showed the *Cry1C* gene transformation did not affect the sensitivity of rice to RDV, indicating *Cry1C* rice would not aggravate the epidemic and dispersal of RDV.

## 1. Introduction

Rice (*Oryza sativa* L.) is one of the most important food crops around the world and serves as a staple food source for more than half of the world’s population [1]. Unfortunately, the yield of rice is often affected by insect pests, leading to great economic loss and a threat to food security [2,3]. The traditional way to reduce the damage caused by insect pests is usually the application of chemical insecticides. Genetic engineering technology provides a cost-effective way to develop transgenic insect-resistant rice plants [4]. A series of transgenic rice lines expressing Cry proteins derived from the soil bacterium *Bacillus thuringiensis* (*Bt*) have been developed to control lepidopteran pests [4,5]. The Chinese government has issued the biosafety certificates of two Cry1Ab/Ac rice lines (Huahui1 and *Bt*-Shanyou 63) [5]. To date, assessments of *Bt* rice on non-target organisms and the environment have been conducted thoroughly, and negligible adverse effects have been found [4,5,6,7,8,9]. However, *Bt* rice is not yet commercially produced largely due to the lack of public acceptance.

Metabolomics is an analytical approach providing an unprejudiced identification and quantification of various metabolite classes in biological materials [10,11,12]. Gas or liquid (GC/LC) chromatography mass spectrometry (MS) based metabolomics has been conducted to detect a wide range of low-molecular-weight metabolites such as amino acids, organic acids, carbohydrates, fatty acids, fatty alcohols, wax esters, sterols, and so on [13,14,15]. In recent years, metabolomic techniques are beginning to be used for assessing intended and unintended changes in genetically modified (GM) crops and conventionally bred crops, as it can provide a whole profile of plant metabolites and could be considered as an alternative supplementary for the safety assessment [16,17,18,19,20,21,22,23]. GC-MS-based metabolic profiling was employed by Zhou et al. [18] to determine the metabolic changes in rice grains. More metabolic changes were found from the tissue culture than those from exogenous gene insertion. Studies of field grown transgenic rice and maize indicated that environmental factors such as sowing dates or locations had a greater influence than gene modification on plant metabolome [16,17]. The seed metabolome of an herbicide-tolerant soybean line SYHT06W had no significant deviation from the natural variation range of metabolome within 48 commercial soybean lines [19]. Grains of carotenoid-biofortified transgenic rice had a similar polar metabolite profiling to its non-transgenic counterpart [20]. The extent of metabolome modification occurring in leaves of genetic engineered rice and maize did not go beyond that in traditional cross-breeding crops [21,23]. Cross with traditional rice cultivars could help to reduce unwanted variation occurring during transformation processes [18,22]. Although most reported studies showed that GM crops had little difference with its non-transgenic parents in the metabolome, differences in metabolic changes were found between transgenic rice and its non-transgenic counterpart in response to insecticide. Insecticide led to much stronger regulations of signaling molecules and antioxidants in Cry1Ab rice B4b87 at the early stage, compared with its non-transgenic counterpart MH86 [24]. Similar results were also reported when comparing the metabolic response to insecticide stress between Cry1Ac/Sck rice 8Km732 and its non-transgenic counterpart MH3301 [25]. Previous studies indicated that agrobacterium-mediated transformation would inevitably stimulate some defense pathways; for example, N-acetylglutamate and phytocassane, which play important roles in plant resistance, were increased in transgenic rice containing *cryIAc-bt* and *G10*-*epsps* genes [22,26,27]. Whether defense-related metabolite changes in *Cry1C* rice, which would change the disease resistance of rice, is largely unknown.

Rice dwarf virus (RDV), the causal agent of rice dwarf disease causing economic damage in many Asian countries, belongs to *Phytoreovirus* in the family *Reoviridae* [28,29,30]. RDV is mainly transmitted by green rice leafhoppers (GRLHs), *Nephotettix cincticeps* (Hemiptera: Cicadellidae) in a persistent-propagative manner. RDV-free GRLHs acquire the virus by feeding on RDV-infected plants for a few minutes or several days. RDV can proliferate in almost 70% of GRLH individuals, which become viruliferous after a latent period of 2–3 weeks [31]. Viruliferous GRLHs could transmit RDV to rice plants throughout their lifetime. RDV infection would cause considerable damage to rice plants such as stunted growth, white chlorotic specks on leaves, incomplete panicle exsertion, resulting in significant yield loss [28,32,33].

In this study, the metabolic profiles of RDV-free or RDV-infected *Bt* rice T1C-19 and its non-*Bt* parental rice MH63 were analyzed using a GC-MS-based metabolomic technique. We compared the metabolic profiles of T1C-19 and MH63 to evaluate the metabolic changes as well as the differences in metabolic response to RDV infection. Free amino acid (AA) contents in rice leaves were investigated to validate the effects of exogenous gene transformation and RDV infection on amino acid metabolism. In addition, the RDV infection rates of the two rice plants were detected. Our results turned out that *Cry1C* gene transformation did not affect the sensitivity of rice plants to RDV. Thus, it would not pose potential environmental risks when *Cry1C* rice plants are exposed to RDV.

## 2. Results

### 2.1. Metabolic Profiling Overview

**Figure 1:**
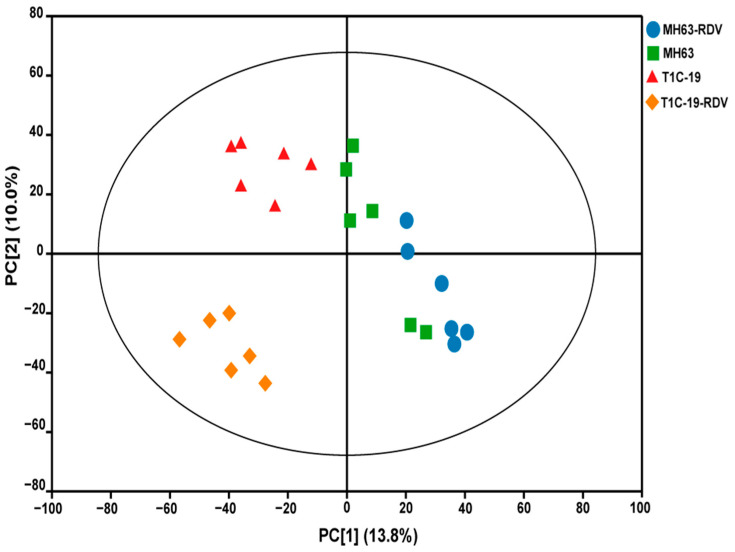
Principal component analysis (PCA) score plots of the 320 identified metabolites in rice leaves of the four treatments. MH63, green square; T1C-19, red triangle; MH63-RDV, blue circle; T1C-19-RDV, orange rhombus.

**Figure 2:**
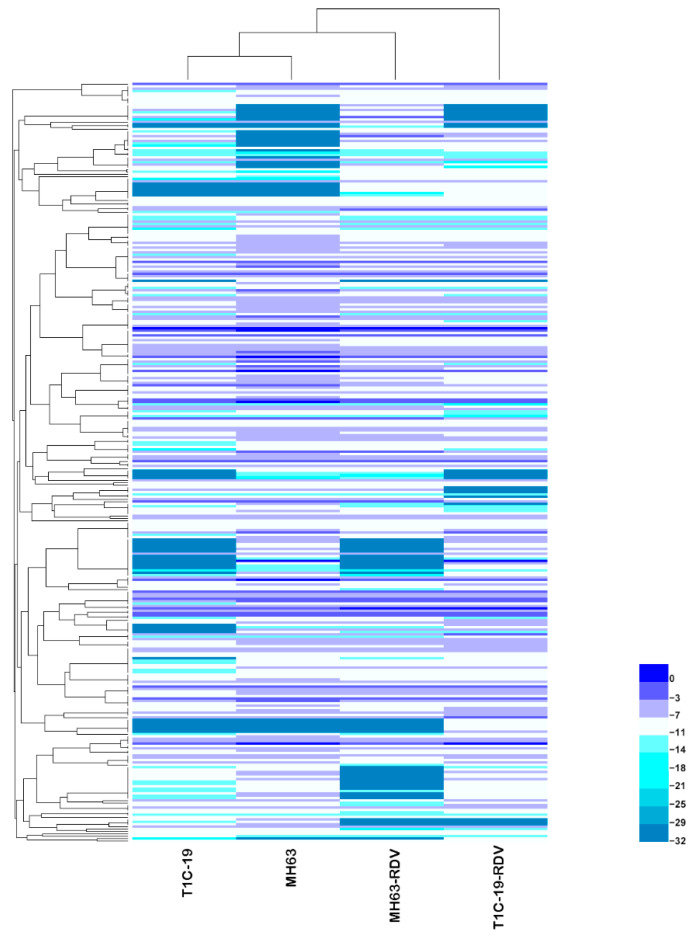
Hierarchical cluster analysis of the 320 identified metabolites in rice leaves of the four treatments. In the heatmap, each treatment is visualized in a single column, and each metabolite is represented by a single row. Metabolite accumulation is shown in different colors, where blue indicates high abundance and low relative expression is shown in indigo (color key scale on the right of the heat map).

**Figure 3:**
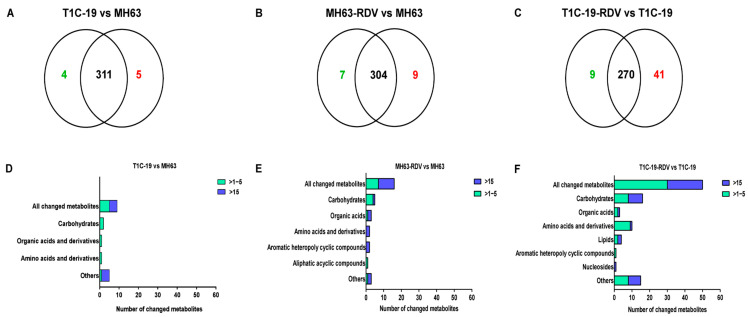
Number of the changed metabolites in three different rice groups. Venn diagrams depicting the changed metabolites between T1C-19 and MH63 rice plants (**A**), between MH63-RDV and MH63 rice plants (**B**), and between T1C-19-RDV and T1C-19 rice plants (**C**). Classification of the changed metabolites and the fold changes between T1C-19 and MH63 rice plants (**D**), between MH63-RDV and MH63 rice plants (**E**), and between T1C-19-RDV and T1C-19 rice plants (**F**).

To understand the effects of *Bt* gene transformation and RDV infection on metabolites in rice leaves, the metabolic profiles of MH63, T1C-19, MH63-RDV, and T1C-19-RDV rice plants were analyzed. A total of 1054 peaks were primordially detected by Gas Chromatography Tandem Time-of-Flight Mass Spectrometry (GC-TOF-MS), and 920 effective peaks were obtained after normalization. Finally, 320 substances including carbohydrates, fatty acids, organic acids, aliphatic acyclic compounds, aromatic heteropoly cyclic compounds, nucleosides, amino acids, and other compounds were identified (Table S1). PCA was conducted using peak numbers and normalized peak areas, which showed a global view of metabolic differences among the samples. As seen in PCA score plots, MH63 and MH63-RDV samples were separated from T1C-19 and T1C-19-RDV on PC1, which accounted for 13.8% of the variability among the samples; MH63-RDV and T1C-19-RDV samples were separated from T1C-19 on PC2, which accounted for 10.0% of the total variability (Figure 1). PLS-DA and permutation tests confirmed the model effectiveness for each compared group (R^2^Y_cum_ > 0.98, Q^2^Y_cum_ between 0.778 and 0.911, Figures S1 and S2). After correction by OPLS-DA, Variable importance in the projection (VIP) of the first principal component (VIP > 1) and Student’s *t*-test (*p* < 0.05) were used to identify the differential metabolites for each compared group. Hierarchical cluster analysis (HCA) of the 320 identified metabolites from the four different rice plants showed that the metabolic profiles of T1C-19 and MH63 clustered more closely, when compared with those of RDV-infected rice plants (MH63-RDV, T1C-19-RDV). The metabolic profile of T1C-19-RDV rice plants showed more differences with the others (Figure 2). Numbers of differential metabolites between each group were summarized in a Venn diagram (Figure 3A–C). Slice metabolite changes (<3% of total metabolites) were predicted between *Bt* rice T1C-19 and MH63 (Figure 3A, Table S2), while much more metabolites were altered by RDV infection in both MH63 and T1C-19 (Figure 3B,C).

### 2.2. Classification of Differential Metabolites between Each Rice Group

The predicted differential metabolites between T1C-19 and MH63 (calculated by log_2_^T1C-19/MH63^) included 3 carbohydrates, 1 amino acid, 2 organic acids, and 3 others (Figure 3D). Gentiobiose, threonic acid, and 2-deoxyerythritol were higher in T1C-19 than in MH63, while cytidine-monophosphate was lower in T1C-19 than in MH63 (Table S2). The 16 metabolites showed significant response to RDV infection in MH63 (calculated by log_2_^MH63-RDV/MH63^) containing 5 carbohydrates, 3 organic acids, 2 amino acids, 1 aliphatic acyclic compound, 2 aromatic heteropoly cyclic compounds, and 3 others (Figure 3E). The accumulation of D-arabitol (29.34-fold), 5-oxoproline (29.06-fold), lactic acid (26.38-fold), threonic acid (21.76-fold), and 2-deoxyerythritol (22.40-fold) was dramatically induced by RDV infection, while the relative concentrations of cytidine-monophosphate (−25.94-fold), glutamine (−25.57-fold), nicotinic acid (−24.45-fold) and acetophenone (−22.54-fold) were reduced. Meanwhile, 50 metabolites were altered in T1C-19-RDV plants when compared with RDV-free T1C-19 plants, including 16 carbohydrates, 10 amino acids and derivatives, 1 nucleoside, 4 lipids, 3 organic acids, 1 aromatic heteropoly cyclic compound, and 15 others (Figure 3F). Metabolites showed a more than 20-fold change (calculated by log_2_^T1C-19-RDV/T1C-19^) containing D-talose (36.2-fold), fructose (28.14-fold), galactinol (27.07-fold), 3,6-anhydro-d-galactose (24.13-fold), glucose-1-phosphate (21.67-fold), palatinitol (21.77-fold), N-acetyl-l-leucine (24.97-fold), uridine (22.40-fold), glutaric acid (25.35-fold), aldosterone (22.43-fold), citraconic acid (25.57-fold), malonamide (25.15-fold), gluconic lactone (23.46-fold), phosphate (23.2-fold), 2-aminophenol (22.12-fold), acetol (21.89-fold), tetracosane (21.61-fold), gentiobiose (−24.30-fold), isomaltose (−24.69-fold), and glutaraldehyde (−22.93-fold). Three significantly changed metabolites were found in all the three comparative analysis groups, including glutamine, threonic acid, and 2-deoxyerythritol (Tables S2–S4), and they showed similar trends in two groups (T1C-19 vs. MH63, MH63-RDV vs. MH63) but opposite to the other (T1C-19-RDV vs. T1C-19). Cytidine-monophosphate showed significant changes with similar trends in groups T1C-19 vs. MH63 and MH63-RDV vs. MH63, while gentiobiose and isomaltose showed significant changes with opposite trends in groups T1C-19 vs. MH63 and T1C-19-RDV vs. T1C-19 (Tables S2–S4).

### 2.3. Pathway Analysis of Differential Metabolites

**Figure 4:**
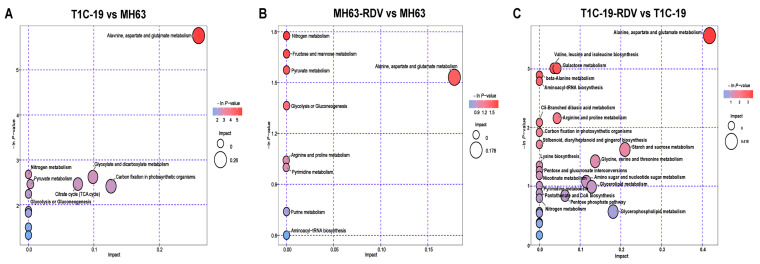
Pathway enrichment of the significantly changed metabolites. Pathway enrichment of the changed metabolites between T1C-19 and MH63 (**A**), between MH63-RDV and MH63 (**B**), and between T1C-19-RDV and T1C-19 (**C**). The abscissa represents the impact of the path topological analysis, and the ordinate is the negative logarithm of *p*-value of the pathway enrichment analysis. The bigger the bubble, the bigger the impact is; the deeper the color, the larger the value of −ln (*p*), indicates the more significant the enrichment.

The pathway analysis of the differential metabolites from three groups is shown in a bubble chart (Figure 4). The predicted differential metabolites between MH63 and T1C-19 were enriched to alanine, aspartate and glutamate metabolism, nitrogen metabolism, carbohydrate metabolism pathways including glycolysis or gluconeogenesis, pyruvate metabolism, TCA cycle, glyoxylate and dicarboxylate metabolism, and energy metabolism pathway carbon fixation (Figure 4A). In response to RDV infection, nitrogen metabolism, carbohydrate metabolism pathways including fructose and mannose metabolism, glycolysis or gluconeogenesis, pyruvate metabolism, and amino acid metabolism pathways including alanine, aspartate and glutamate metabolism, and arginine and proline metabolism were significantly changed in MH63 (Figure 4B). Compared with MH63, other amino acid metabolism pathways were more affected by RDV infection in T1C-19, such as valine, leucine and isoleucine biosynthesis, beta-alanine metabolism, aminoacyl-tRNA biosynthesis, lysine biosynthesis and glycine, and serine and threonine metabolism. The biosynthesis of other secondary metabolites (stilbenoid, diarylheptanoid, and gingerol) and lipid metabolism pathways showed significant response to RDV infection in T1C-19, as well as cofactors and vitamin metabolism pathways (Figure 4C). Metabolites involved in carbohydrate metabolism pathways also showed significant changes in response to RDV infection in T1C-19, but they were different with those in MH63. 

### 2.4. Effects of Rice Type and RDV Infection on Free Amino Acids (AAs) Contents in Rice Leaf Tissues

Considering the changes in amino acid metabolism pathways between rice types, as well as between RDV-infected and RDV-free plants, the free AA contents in rice leaves were determined. Concentrations of threonine (Thr), valine (Val), and isoleucine (Ile) were a little bit higher in T1C-19 than in MH63 (Table 1). However, no significant difference in free AA contents was found between rice types. In contrast, concentrations of AAs were increased after RDV infection, and significant effects were detected for 11 AAs including Thr, serine (Ser), alanine (Ala), cysteine (Cys), Val, Ile, leucine (Leu), phenylalanine (Phe), gamma aminobutyric acid (g-ABA), lysine (Lys), and arginine (Arg) (Table 1). The interaction of rice type and RDV infection had no significant effects on free AA contents in rice plants expect for Ala and Leu (Table 1).

### 2.5. The RDV Infection Rates between Cry1C and MH63 Rice Plants

**Figure 5:**
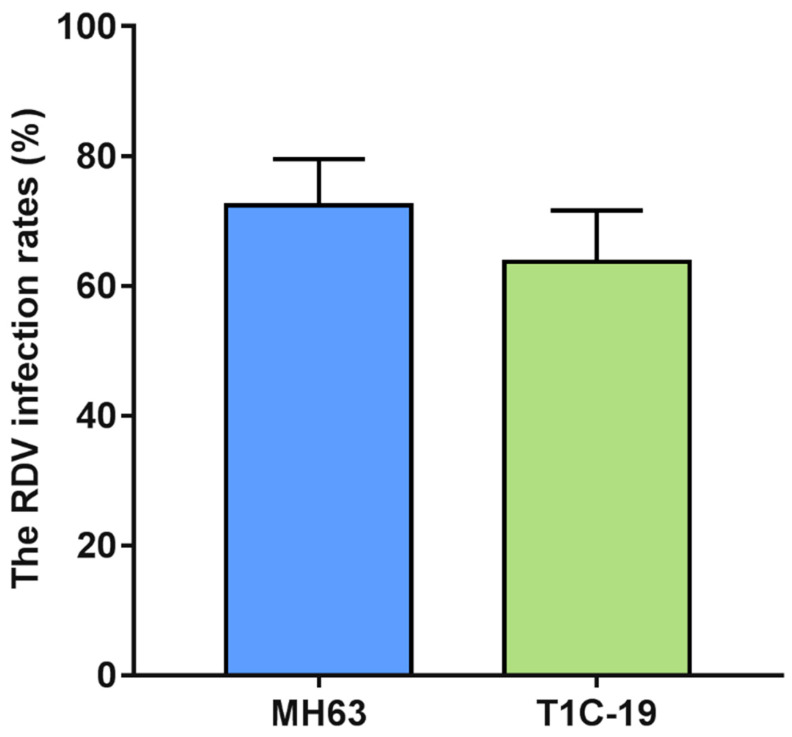
The RDV infection rates between *Cry1C* and MH63 rice plants. The RDV infection rates were analyzed using Student’s *t*-test. Values are mean ± standard error (n = 3).

The RDV infection rates in MH63 and *Cry1C* rice plants were 72.38% and 63.72%, and no significant difference was found between *Cry1C* and MH63 rice plants (*t* = 0.812, *df* = 4, *p* = 0.462) (Figure 5).

## 3. Discussion

Omics techniques such as transcriptomics, proteomics, and metabolomics provide powerful approaches for analyzing the unintended changes in genetically engineered plants [21,23,34,35,36,37,38]. The transcriptomes, proteomes, or metabolomes of different *Bt* rice events were compared with their non-transgenic parents and other traditional-breeding rice cultivars [17,18,23,39]. It was reported that, compared with tissue culture, environmental factors and natural variations among rice cultivars with different genetic backgrounds, transformations, and exogenous gene insertions had much less influence on the rice transcriptome, proteome, or metabolome [17,18,23,39].

The present work compared the metabolic profiles between *Bt* rice T1C-19 and its non-*Bt* parental rice MH63, as well as those between RDV-infected and RDV-free rice. Less than 3% of total metabolites showed difference between T1C-19 and MH63 rice, which were far less than those between traditional rice varieties (e.g., 8.26–33.94% in Fu et al. [22]; 10.08–26.91% in Liu et al. [23]), while 16 (5.0%) and 50 (15.62%) compounds showed significant changes in response to RDV infection in MH63 and T1C-19, respectively (Figure 3). The results indicated that *Cry1C* gene insertion and transformation had negligible impact on the metabolome of rice leaves, which was consistent with previous studies [22,23,27]. Similar results have also been reported in maize and barley [16,21,40]. A recent study reported lower phloem transport capacity of potassium to grain and worse growth performance in *Cry1C* rice T1C-19 than in MH63 by pot experiments with different levels of K fertilizer [41]. They inferred it might be due to unintended changes caused by *Cry1C* gene insertion. However, Fu and Liu [42] reported that T1C-19 had a stronger reproductive capacity than MH63 when grown in saline-alkaline soil. No related change was found in T1C-19 at either transcriptomic or metabolomic level based on the present and previous studies [23]. What caused such variation needs to be further studied.

Similar as that of insecticide treatment, RDV infection had a greater influence on rice metabolic profiling, especially on metabolites in pathways involving metabolism of carbohydrates, amino acids and derivatives, and organic acids and derivatives [24,25]. Some aromatic, heteropoly cyclic, and aliphatic cyclic compounds were significantly affected by RDV infection, but they were not consistent in the two rice lines. Four metabolites in MH63 showed similar response to RDV infection with that to *Cry1C* gene transformation (T1C-19 vs. MH63), including glutamine, threonic acid, 2-deoxyerythritol, and cytidine-monophosphate (Tables S2–S4). It was supposed that these might be compounds related to stimuli response. Previous studies have revealed that the process of foreign gene insertion was somewhat like that of exposure to biotic or abiotic stress, as genes involved in the plant immunity signaling pathways showed significant changes [22,26].

When comparing the effects of RDV infection on metabolomes between rice lines, carbohydrate metabolism and amino acid metabolism pathways showed more significant response to RDV in T1C-19 than those in MH63. Some lipid metabolism related compounds and secondary metabolites (such as resveratrol, hesperitin and farnesol, classified into the others) showed significant response to RDV infection in T1C-19 ( Figure 3; Figure 4; Tables S2–S4). Similar with our results, insecticide led to much stronger regulations of ascorbate, α-tocopherol, and the shikimate-mediated secondary metabolism in *Bt*-transgenic variety B4b87 at the early stage. More flavonoids were significantly changed in transgenic rice [24,25]. Although some differences in the metabolome and metabolic response to RDV infection did exist between T1C-19 and MH63, the RDV infection rates had no difference between the two rice lines (Figure 5).

Metabolic changes involved in the amino acid metabolism pathway have been reported in a few *Bt* rice events [18,26,27]. In the present study, free amino acid contents in *Bt* rice T1C-19 had no significant difference with its non-*Bt* parental rice MH63, although differentially accumulated metabolites were enriched in alanine, aspartate, and glutamate metabolism pathways. Meanwhile, the contents of free amino acids were induced by RDV infection. Seven of them were significantly higher in RDV-infected rice plants than in RDV-free rice, including Ala, Val, Ile, Leu, Phe, g-ABA, and Lys, which might be conductive to improving the performance of insect vectors feeding on virus-infected plants [43,44,45]. However, no more changes in amino acid contents in response to RDV infection were found in the transgenic rice T1C-19, which was different with the results of metabolic profiling analysis (Table 1, Figure 4). We inferred that some amino acid metabolism pathways might be associated with stress response, but not amino acid biosynthesis.

## 4. Materials and Methods

### 4.1. Insects and Plants

The GRLH colonies were collected from the paddy field at the experiment farm of Zhejiang University, Hangzhou, China. The colony was maintained on the susceptible rice “Taichung Native1” (TN1) seedlings covered with a nylon cage (80-mesh, 50 cm × 50 cm × 50 cm) for 3–4 generations. It was kept in a climate chamber at 27 ± 1 °C, 75 ± 5% relative humidity, under a photoperiod of 14 h: 10 h (light: dark). Viruliferous GRLHs were obtained as described previously [9]. Non-viruliferous GRLH nymphs were confined with RDV-infected TN1 rice plants for 48 h and then transferred into RDV-free TN1 rice plants. About two weeks later, these nymphs were individually released into plastic tubes (diameter 2.5 cm, height 25 cm) with one seedling in each tube and marked. Rice seedlings were replaced every two days, and these seedlings were transplanted in the greenhouse with its reference number. Viruliferous GRLHs were collected as typical RDV symptoms appeared on relative rice plants and reared on the RDV-infected plants under the same condition as described above until the experiment.

*Bt* rice line T1C-19 (*Cry1C* rice) was generated by Tang et al. [46] through the agrobacterium-mediated transformation of the synthetic *Cry1C* gene into a widely used restorer rice line Minghui63 (MH63). The 14th selfing progeny of the homozygous T1C-19 and the relative non-*Bt* parental rice MH63 were used in this study. To obtain RDV-infected rice plants, 4th or 5th instar viruliferous GRLH nymphs were transferred to plastic tubes with rice seedlings (10 days) individually after 2 h starvation. For consistency, RDV-free rice seedlings were fed by non-viruliferous GRLHs nymphs. After 48 h of treatments, all the GRLHs were removed, and rice seedlings were transplanted into a greenhouse hydroponically under natural lighting at the temperature of 25 ± 2 °C. The RDV infection status of the rice plants was confirmed by RT-PCR [47]. A total of 60 individual rice plants were prepared for each treatment (MH63, T1C-19, MH63-RDV, T1C-19-RDV) for metabolite profiling analysis and free amino acid content analysis.

### 4.2. Metabolite Extraction and Metabolite Profiling Analysis

Metabolite extraction and metabolite profiling analyses were similar as Guo et al. [48]. Approximately 50 mg rice leaf tissue (a mixture of three flag leaves from three 45-old-day rice plants) for each sample was weighted into a 2 mL tube. Then, 0.4 mL extraction buffer (methanol: chloroform at a volume ratio of 3:1) and 20 μL of ribitol (0.2 mg ml^−1^ stock in ddH_2_O, Sigma-Aldrich, Saint Louis, MO, USA) as an internal standard were added into this tube. After vortex mixing for 10 s, the samples were homogenized in a ball mill (Jinxin Biotech LTD. Shanghai, China) at 55 Hz for 5 min. The mixtures were centrifuged at 12,000 rpm at 4 °C for 15 min, then 0.4 mL of supernatant was transferred into a new 2 mL GC-MS sample vial and dried in a vacuum concentrator at 30 °C for 1.5 h. For derivatization, first, 80 μL methoxyamine (20 mg mL^−1^, dissolved in pyridine) was added and incubated at 80 °C for 20 min after mixing and sealing. Second, 100 μL BSTFA (containing 1% TCMS, *v*/*v*, Regis Technologies, Morton Grove, IL, USA) was added for trimethylsilylation and incubated at 70 °C for 1 h. Six independent replications were prepared for each treatment.

GC-TOF-MS analysis was performed using Agilent 7890 gas chromatograph system (Agilent Technologies, Santa Clara, CA, USA) coupled with a Pegasus HT time-of-flight mass spectrometer (LECO Corporation, Saint Joseph, MI, USA). DB-5 MS capillary column coated with 5% diphenyl cross-linked with 95% dimethyl polysiloxane (30 m × 250 μm inner diameter, 0.25 μm film thickness; J & W Scientific, Folsom, CA, USA) was used to separate metabolites. An aliquot of 1 μL derivative extract was injected in splitless mode with the carrier gas helium. The front inlet purge flow was 3 mL min^−1^, and the gas flow rate through the column was 1 mL min^-1^. The initial column temperature was kept at 50 °C for 1 min, 10 °C min^−1^ rate up to the target of 330 °C, and then held for 5 min. The injection, transfer line, and ion source temperatures were set at 280, 280, and 220 °C, respectively. The ionization voltage was −70 eV in electron impact mode. Spectra were recorded in full-scan mode with a scanning range from 85 to 600 m/z, at a rate of 20 spectra per second after a solvent delay of 366 s.

### 4.3. Analysis of Free Amino Acid (AA) Contents

Approximately 1 g fresh rice leaf tissue (a mixture of six flag leaves from six 45-old-day rice plants) of each sample was collected and grinded in the mortar with liquid nitrogen. Then the powder was transferred to a 10 mL centrifuge tube containing 3% sulfosalicylic acid solution and oscillated for 1 h. After centrifuging at 10,000 rpm at 4 °C for 15 min, the supernatant was collected and filtered through 0.45 μm pore size membrane filter for AA content analysis. Aliquots (20 μL) of the supernatants for each sample were injected into an automated amino acid analyzer (L-8900, Hitachi, Japan) [49]. Five independent replications for each treatment (MH63, T1C-19, MH63-RDV and T1C-19-RDV) were tested.

### 4.4. The RDV Infection Rates between Cry1C and MH63 Rice Plants

A group of 40 viruliferous GRLHs were selected and individually transferred into plastic tubes (one rice seeding in each tube). Three biological replications were used for studying the RDV infection rates between *Cry1C* and MH63 rice plants. Rice seedlings were collected after 48 h GRLHs feeding and transplanted to a greenhouse with the same method as described above. Fifteen days later, the RDV infection status of *Cry1C* and MH63 rice plants were determined by RT-PCR [47], and the RDV infection rates were calculated.

### 4.5. Data Processing and Statistical Analysis

Chroma TOF 4.3X software of LECO Corporation and LECO-Fiehn Rtx5 database were used for raw peak extraction, peak alignment, peak identification, and integration of the peak area [50]. Retention time index (RI) was used for peak identification, and the RI tolerance was 5000. The resultant three-dimensional data, including peak numbers, sample names, and normalized data, were then imported into the “soft independent modelling of class analogy” (SIMCA) 13.0 software (Umetrics, Umea, Sweden) for principal component analysis (PCA), partial least-squares discriminant analysis (PLS-DA), and orthogonal PLS discriminant analysis (OPLS-DA). A hierarchical clustering heat map was created using HemI (1.0.3.7) software. A two-step strategy was used to calculate the distance among treatments. The distance between data sets was calculated using ‘Pearson distance’, while distance between clusters was calculated by ‘Average linkage clustering’ algorithm [51]. Variable importance in the projection (VIP > 1) of the first principal component using the OPLS-DA model and Student’s *t*-test (*p* < 0.05) were used to identify the differential metabolite. The three compared groups were T1C-19 vs. MH63, MH63-RDV vs. MH63, and T1C-19-RDV vs. T1C-19. Discriminatory compounds were then mapped to metabolic pathways in MetaboAnalyst (https://www.metaboanalyst.ca), while KEGG (Kyoto Encyclopaedia of Genes and Genomes) was selected for database search. Fisher exact test was selected for enrichment, and relative betweenness centrality was chosen for topology analysis [52].

Data on free AA contents of rice leaves and the RDV infection rates were analyzed using SPSS 20.0 software. The free AA contents of four different groups were analyzed using general linear models (GLMs) followed by Tukey’s multiple range test. The RDV infection rates between *Cry1C* and MH63 rice plants were analyzed using Student’s *t*-test.

## 5. Conclusions

In summary, we compared the metabolic profiling of *Bt* rice T1C-19 with its non-*Bt* parent rice MH63 and also the differences in metabolic changes in response to RDV stress between the two rice lines. Slice difference was detected in metabolic profiles between T1C-19 and MH63, while RDV infection had an obviously greater influence on metabolic profiles of both rice lines. We should be aware that metabolite changes alone do not mean it is unsafe. As it has been found in this study, the transformation of *Cry1C* gene does not affect the RDV sensitivity of rice plants. The greenhouse experiments can reflect the field experiments to a certain extent, due to the complex environmental factors in the field. To further validate the results in the greenhouse, field experiments are the direction of our next research.

## Figures and Tables

**Table 1:** Effects of rice type, RDV infection status, and the interaction of rice type and RDV infection status on free amino acid content in rice plants.

Content (μg/g)	MH63	MH63-RDV	T1C-19	T1C-19-RDV	Two-Way ANOVA
Aspartic acid (Asp)	142.286 ± 23.315 a	183.935 ± 23.135 a	106.239 ± 20.036 a	159.286 ± 23.135 a	*F_A_* = 1.835, *p* = 0.209; *F_B_* = 4.468, *p* = 0.064; *F_A × B_* = 0.065, *p* = 0.805
Threonine (Thr)	90.659 ± 19.691 b	160.652 ± 19.691 ab	109.088 ± 17.052 ab	176.621 ± 19.691 a	*F_A_* = 0.814, *p* = 0.391; *F_B_* = 13.008, *p* = 0.006; *F_A × B_* = 0.004, *p* = 0.950
Serine (Ser)	144.014 ± 18.571 a	188.826 ± 18.571 a	161.652 ± 16.083 a	216.211 ± 18.571 a	*F_A_* = 1.567, *p* = 0.242; *F_B_* = 7.636, *p* = 0.022; *F_A × B_* = 0.073, *p* = 0.792
Glutamic acid (Glu)	287.509 ± 60.978 a	380.280 ± 60.978 a	298.339 ± 52.809 a	191.462 ± 60.978 a	*F_A_* = 2.272, *p* = 0.166; *F_B_* = 0.014, *p* = 0.908; *F_A × B_* = 2.859, *p* = 0.125
Glycine (Gly)	26.859 ± 3.578 a	26.632 ± 3.578 a	30.171 ± 3.099 a	29.133 ± 3.578 a	*F_A_* = 0.704, *p* = 0.423; *F_B_* = 0.033, *p* = 0.859; *F_A × B_* = 0.014, *p* = 0.909
Alanine (Ala)	96.375 ± 11.656 b	192.164 ± 11.656 a	112.280 ± 10.094 b	125.361 ± 11.656 b	*F_A_* = 5.085, *p* = 0.051; *F_B_* = 23.266, *p* = 0.001; *F_A × B_* = 13.427, *p* = 0.005
Cysteine (Cys)	26.226 ± 4.262 a	36.919 ± 4.262 a	33.514 ± 3.691 a	42.092 ± 4.262 a	*F_A_* = 2.280, *p* = 0.165; *F_B_* = 5.453, *p* = 0.044; *F_A × B_* = 0.066, *p* = 0.803
Valine (Val)	14.860 ± 2.457 b	25.592 ± 2.457 a	17.348 ± 2.127 ab	20.525 ± 2.457 ab	*F_A_* = 0.294, *p* = 0.601; *F_B_* = 8.547, *p* = 0.017; *F_A × B_* = 2.522, *p* = 0.147
Methionine (Met)	332.581 ± 38.217 a	341.890 ± 38.217 a	367.295 ± 33.097 a	311.576 ± 38.217 a	*F_A_* = 0.004, *p* = 0.954; *F_B_* = 0.393, *p* = 0.546; *F_A × B_* = 0.772, *p* = 0.402
Isoleucine (Ile)	5.215 ± 0.612 c	8.192 ± 0.612 ab	5.667 ± 0.530 bc	9.044 ± 0.612 a	*F_A_* = 1.213, *p* = 0.299; *F_B_* = 28.769, *p* < 0.001; *F_A × B_* = 0.114, *p* = 0.743
Leucine (Leu)	15.582 ± 2.095 b	17.954 ± 2.095 b	13.859 ± 1.815 b	27.198 ± 2.095 a	*F_A_* = 3.436, *p* = 0.097; *F_B_* = 14.992, *p* = 0.004; *F_A × B_* = 7.306, *p* = 0.024
Tyrosine (Tyr)	26.524 ± 3.563 a	25.708 ± 3.563 a	28.631 ± 3.086 a	33.962 ± 3.563 a	*F_A_* = 2.255, *p* = 0.167; *F_B_* = 0.428, *p* = 0.529; *F_A × B_* = 0.794, *p* = 0.396
Phenylalanine (Phe)	18.635 ± 1.687 b	27.756 ± 1.687 a	17.272 ± 1.461b	28.749 ± 1.687 a	*F_A_* = 0.013, *p* = 0.912; *F_B_* = 39.741, *p* = 0.000; *F_A × B_* = 0.520, *p* = 0.489
Gamma aminobutyric acid (g-ABA)	213.114 ± 39.964 b	418.288 ± 39.964 a	232.886 ± 34.610b	466.853 ± 39.964 a	*F_A_* = 0.78, *p* = 0.400; *F_B_* = 32.198, *p* < 0.001; *F_A × B_* = 0.138, *p* = 0.718
Lysine (Lys)	23.069 ± 3.050 b	34.497 ± 3.050 ab	24.039 ± 2.641 b	39.426 ± 3.050 a	*F_A_* = 0.998, *p* = 0.344; *F_B_* = 20.612, *p* = 0.001; *F_A_*_× *B*_ = 0.449, *p* = 0.519
Histidine (His)	6.890 ± 0.699 a	8.511 ± 0.699 a	6.650 ± 0.605 a	7.877 ± 0.699 a	*F_A_* = 0.416, *p* = 0.535; *F_B_* = 4.429, *p* = 0.065; *F_A_*_× *B*_ = 0.085, *p* = 0.777
Arginine (Arg)	15.512 ± 2.206 a	23.356 ± 2.206 a	18.116 ± 1.911 a	22.678 ± 2.206 a	*F_A_* = 0.203, *p* = 0.663; *F_B_* = 8.433, *p* = 0.017; *F_A × B_* = 0.590, *p* = 0.462

Note: The data are presented as means ± standard error (*n* = 5). Four groups of free amino acid content were analyzed using general linear models (GLM) followed by Tukey’s multiple range test. Different letters in the same row indicate significant differences among treatments (*p* < 0.05). *F_A_*: rice type; *F_B_*: RDV infection status; *F_A × B_*: the interaction of rice type and RDV infection status.

## Data Availability

The data presented in this study are available in the Supplementary Materials.

## Supplementary Materials

The following are available online at https://www.mdpi.com/article/10.3390/metabo11040209/s1, Figure S1. Partial least squares discriminant analysis (PLS-DA) of metabolites for three compared groups. PLS-DA score plots for T1C-19 (green square) and MH63 (red triangle) (A), for MH63-RDV (blue circle) and MH63 (red triangle) (B), for T1C-19-RDV (blue circle) and T1C-19 (green square) (C). Figure S2. Permutation test of PLS-DA for the three compared groups. Two hundred permutations were performed, and the resulting R2 and Q2 values were plotted. (R2, green triangle), (Q2, blue square). The green dots represent the regression line for R2 and the blue dots for Q2. (A) T1C-19 and MH63, (B) MH63-RDV and MH63, (C) T1C-19-RDV and T1C-19. Table S1. Metabolite profiling for compounds in rice leaves identified using mass spectrometry database. Table S2. The relative abundance of differential metabolites in rice leaves by pairwise comparison between T1C-19 and MH63. Table S3. The relative abundance of differential metabolites in rice leaves by pairwise comparison between MH63-RDV and MH63. Table S4. The relative abundance of differential metabolites in rice leaves by pairwise comparison between T1C-19-RDV and T1C-19. Table S5. The raw data of all metabolites. Table S6. The relative contents of all metabolites.
