# Supplementary figures and images for: Metabolic Analysis Reveals Cry1C Gene Transformation Does Not Affect the Sensitivity of Rice to Rice Dwarf Virus
Source: Metabolites. 2021 Mar 30;11(4):209. doi: 10.3390/metabo11040209 (PMC8065979; doi:10.3390/metabo11040209)

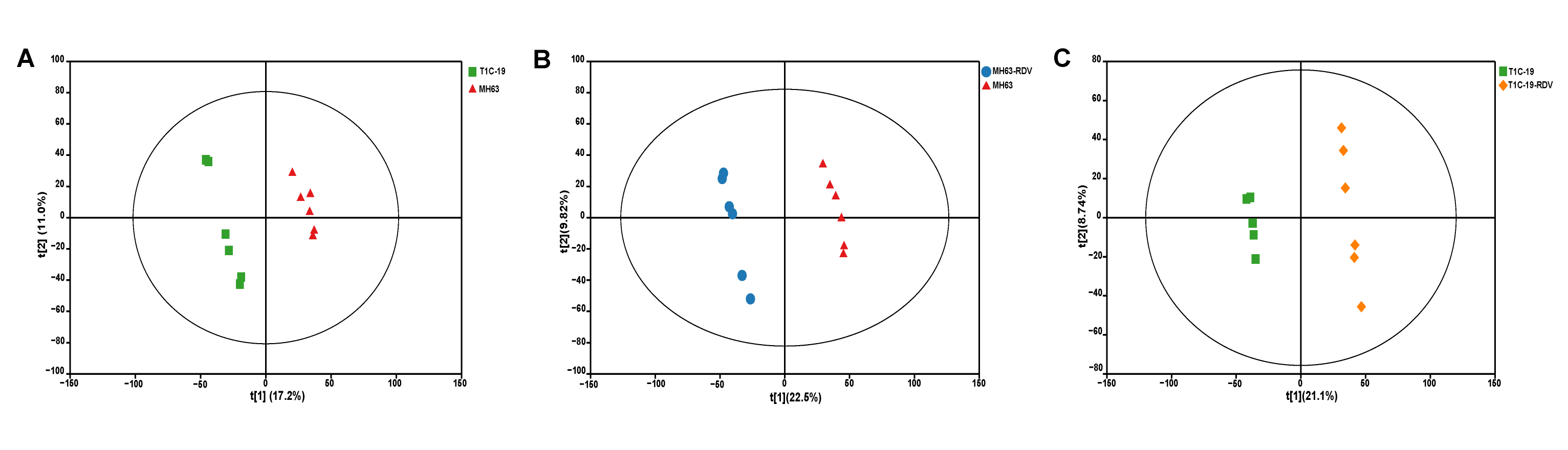

Supplement: Supplementary file 1 [file metabolites-11-00209-s001.zip › Figure S1.tif]

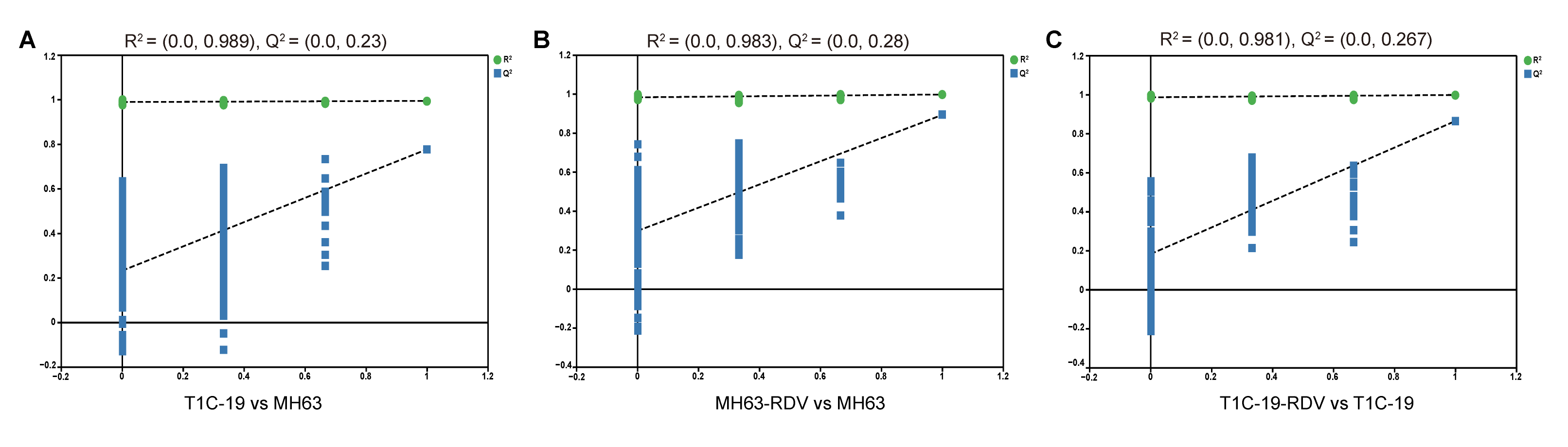

Supplement: Supplementary file 1 [file metabolites-11-00209-s001.zip › Figure S2.tif]
